# Candidate Gene Association Studies in Atopic Dermatitis in Participants of European and Asian Ancestry: A Systematic Review and Meta-Analysis

**DOI:** 10.3390/genes14071456

**Published:** 2023-07-17

**Authors:** Alexandros Pontikas, Charalabos Antonatos, Evangelos Evangelou, Yiannis Vasilopoulos

**Affiliations:** 1 Laboratory of Genetics, Section of Genetics, Cell Biology and Development, Department of Biology, University of Patras, 26504 Patras, Greece; alexandrospontikas@gmail.com (A.P.); charisantonatos@gmail.com (C.A.); 2 Department of Hygiene and Epidemiology, University of Ioannina Medical School, 45110 Ioannina, Greece; 3 Biomedical Research Institute, Foundation for Research and Technology-Hellas, 45110 Ioannina, Greece; 4 Department of Epidemiology & Biostatistics, School of Public Health, Imperial College London, London W2 1PG, UK

**Keywords:** atopic dermatitis, meta-analysis, eczema, association study, polymorphism, SNP, candidate gene

## Abstract

Atopic dermatitis (AD) has been extensively investigated for genetic associations utilizing both candidate gene approaches and genome-wide scans. Here, we comprehensively evaluated the available literature to determine the association of candidate genes in AD to gain additional insight into the etiopathogenesis of the disease. We systematically screened all studies that explored the association between polymorphisms and AD risks in cases of European and Asian ancestry and synthesized the available evidence through a random-effects meta-analysis. We identified 99 studies that met our inclusion/exclusion criteria that examined 17 candidate loci in Europeans and 14 candidate genes in Asians. We confirmed the significant associations between *FLG* variants in both European and Asian populations and AD risk, while synthesis of the available data revealed novel loci mapped to *IL18* and *TGFB1* genes in Europeans and *IL12RB1* and *MIF* in Asians that have not yet been identified by genome-wide association studies. Our findings provide comprehensive evidence for AD risk loci in cases of both European and Asian ancestries, validating previous associations as well as revealing novel loci that could imply previously unexplored biological pathways.

## 1. Introduction

Atopic dermatitis (AD) is a common, chronic inflammatory cutaneous disease characterized by the development of recurrent eczematous lesions and intense itching [1]. AD affects about 20% of the worldwide infant population, displaying a lower prevalence during adulthood, incorporating both persistent as well as new clinical cases [1]. The increasing prevalence, nevertheless, of AD in the industrialized societies depicts the complex interactions of the stable genetic background with the constant perturbations of the environmental factors; AD is considered as a multifactorial disorder with a strong genetic background, accounting for approximately 75% of the total variability [2]. Numerous attempts have been conducted in order to decipher the polygenic etiology of AD, examining both the inter-individual variation of AD cases [3,4] as well as under the atopic march spectrum [5], including asthma, hay fever and AD. The majority of the associated loci lie in the central pathophysiological features of AD, characterized by the epidermal barrier dysfunction and the increased T helper (T_H_) 2 cell-like inflammatory pattern in the skin [1]. Specifically, loss-of-function mutations in the filaggrin (*FLG*) gene, including the rs558269137 (2282del4) and rs61816761 (R501X) [6] Single-Nucleotide Polymorphisms (SNPs), which facilitates the formation of the cornified envelope, have been consistently associated with an increased risk for AD in various ethnic populations, due to their implication in the reduced granular layer [7]. Disease progression is additionally promoted by the participation of microbial alterations and physical damage [1], leading to the increased epithelial permeability and initiation of a type 2 immune response [1] mediated by the TSLP cytokine [8]. Genetic variants mapped in the type 2 cytokine cluster, such as the *IL4* rs2243248 [9] and the *IL13* rs1800925 [10] SNPs, regulate the secretion of several T_H_2-related interleukins (ILs), with the major examples of the *IL4* and *IL13* that have been further targeted by modern therapeutic approaches [11].

Accumulating evidence from genome-wide association studies (GWASs) in participants of European ancestry (*n* = 116,863, *n*_cases_ = 21,399) has identified 31 genetic variants that account for less than 20% of the general heritability [3], while extended rare variant approaches have extended the explained variation by 12.56% [4]. Contrastingly, the latest Asian GWAS reported four novel Asian-specific AD susceptibility loci from a total of 118,287 participants (*n*_cases_ = 2639) [12]. However, discrepancies observed between the number of genome-wide scans conducted in both populations [13] limit the representation of diverse ethnic backgrounds in GWAS. In light of these high-throughput approaches, re-evaluation of the candidate gene studies and gene-by-interaction hypotheses is of paramount importance to complement the above results and further characterize the genetic architecture of complex disorders through genotyping of targeted biological pathways. Nevertheless, the relatively small sample size, lack of statistical power and potentially biased reportage of the derived results hinder the conducting of such approaches despite their established efficacy [14,15,16,17,18,19,20,21,22,23,24,25,26,27,28,29,30,31,32,33,34,35,36,37,38,39,40,41,42,43,44,45,46,47,48,49,50,51,52,53,54,55,56,57,58,59,60,61,62,63,64,65,66,67,68,69,70,71,72,73,74,75,76,77,78,79,80,81,82,83,84,85,86,87,88,89,90,91,92,93,94,95,96,97,98,99,100,101,102,103,104,105,106,107,108,109,110,111,112].

To address this issue, we assessed the inter-individual variability present in the genetic predisposition of AD, incorporating a conservative random-effects meta-analysis (REM) approach to synthesize the evidence derived from available studies and thus unveil putative risk loci associated with the disease onset in participants of European [14,15,16,17,18,19,20,21,22,23,24,25,26,27,28,29,30,31,32,33,34,35,36,37,38,39,40,41,42,43,44,45,46,47,48,49,50,51,52,53,54,55,56,57,58,59,60,61,62,63,64,65,66] and Asian [67,68,69,70,71,72,73,74,75,76,77,78,79,80,81,82,83,84,85,86,87,88,89,90,91,92,93,94,95,96,97,98,99,100,101,102,103,104,105,106,107,108,109,110,111,112] ancestry.

## 2. Materials and Methods

### 2.1. Search Strategy and Selection Criteria

We conducted a systematic literature search to identify studies that examined the inter-individual genetic variability in patients with AD. Searches in the Medline database (through PubMed) using keywords referring to the association (‘association,’ ‘susceptibility’) of genetic variants (‘polymorphism,’ ‘variant,’ ‘SNP,’ and related terms) with the AD trait (‘atopic dermatitis,’ ‘eczema’) from inception to 18 September 2022. Screening was performed by two independent authors in a standardized manner, following relevant titles and abstract reading before full-text reviewing. We considered all case-control candidate-gene studies that examined the genetic predisposition of atopic dermatitis through targeted genotyping, regardless of the age of onset and the allergic disease spectrum that includes hay fever, rhinitis and asthma. Studies included in our analysis were published in peer-reviewed journals in the English language; variants incorporated in our meta-analysis should be investigated by at least two eligible studies. We excluded case reports, abstracts, animal studies, duplicate reported data and reports that did not provide the appropriate data for the calculation of effect size and confidence intervals.

### 2.2. Data Extraction

Two authors extracted concurrently and independently the eligible studies and extracted the appropriate data; discrepancies were resolved through re-examination of the respective publications until consensus was reached. Data extracted from the eligible studies referred to the first author, publication year, total sample size and the reported genotypes for further effect size computations.

Odds Ratios (ORs), along with their 95% Confidence Intervals (95% Cis), were calculated for the allelic model of inheritance. To avoid the parallel assessment of non-independent SNPs in the same locus, we measured linkage disequilibrium (LD) using the LDpop tool provided by the National Institutes of Health (NIH; https://ldlink.nci.nih.gov/?tab=ldpop; accessed on 15 November 2022). R-squared values greater than 0.9 highlighted SNPs in high LD and were therefore considered as a single locus in the meta-analysis.

### 2.3. Statistical Analysis

We calculated OR and 95% CI for each respective heritance pattern by synthesizing the available study-specific evidence through the random-effects model. The presence of heterogeneity was assessed with the Cochran’s Q test (considered significant at *p*-value < 0.1) and quantified via the I^2^ metric, with 0% < I^2^ < 25% reporting small, 25% < I^2^ < 50% reporting moderate, 50% < I^2^ < 75% reporting high and I^2^ > 75% reporting very high heterogeneity. The predominant role of the *FLG* variants in the AD predisposition led us to further evaluate their combined effect due to their similar biological effects. To explore the inter-study variability, we employed the Harbord’s modified test, a robust statistical analysis for the assessment of small-study effects in each meta-analysis. All statistical analyses were performed with the Stata 13.1 software (Stata Corp, College Station, TX, USA). The metan plugin was incorporated for our REMs, while the metabias plugin was utilized for the Harbord’s modified test. Statistical significance threshold was set at *p*-value ≤ 0.05, while the *p*-value < 0.1 threshold was used for the Harbord’s modified test.

## 3. Results

### 3.1. Studies Included in Our Analysis

**Figure 1:**
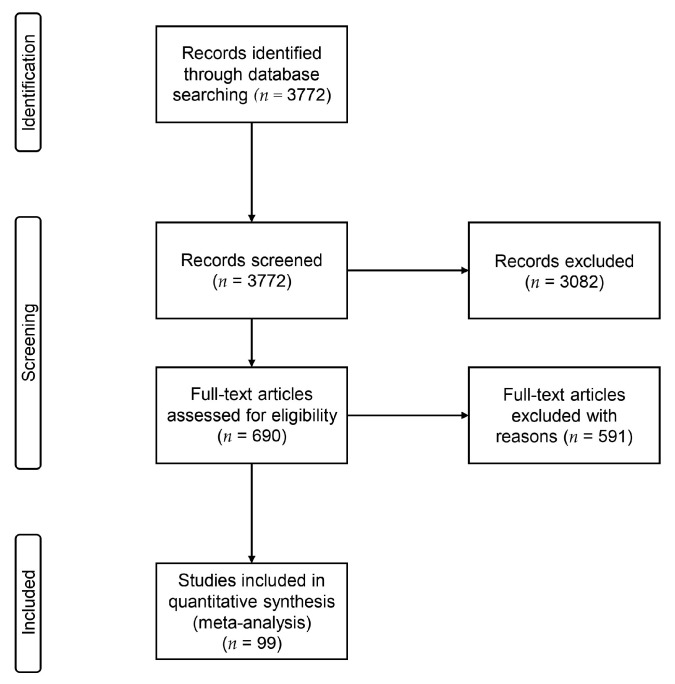
Flow diagram of our included studies.

Our systematic search identified 3772 studies in the PubMed database. During our screening process, 3082 were excluded according to both the abstract and inclusion/exclusion criteria, leaving 690 to be thoroughly assessed. Finally, a total of 99 eligible studies met our predefined criteria and were included in the meta-analysis. A brief overview of the information flow is depicted in Figure 1.

Selected characteristics of the available literature are provided in Table 1 and Table 2. In particular, 53 out of 99 studies (54%) examined participants of European ancestry [14,15,16,17,18,19,20,21,22,23,24,25,26,27,28,29,30,31,32,33,34,35,36,37,38,39,40,41,42,43,44,45,46,47,48,49,50,51,52,53,54,55,56,57,58,59,60,61,62,63,64,65,66] (Table 1) and 46 (46%) of Asian ancestry [67,68,69,70,71,72,73,74,75,76,77,78,79,80,81,82,83,84,85,86,87,88,89,90,91,92,93,94,95,96,97,98,99,100,101,102,103,104,105,106,107,108,109,110,111,112] (Table 2). Studies conducted in European participants focused on the *FLG* [14,15,16,17,18,19,20,21,22,23,24,25,26,27,28,29,30,31,32,33,34,35,36,37,38,39,40] (*n* = 27, 27%), *TLR2* [41,42,43,44,45,46,47] (*n* = 7, 7%), *IL10* [48,49,50,51,52,53,54] (*n* = 7, 7%), 11q13.5 [25,40,55,56,57] (*n* = 5, 5%), *IL13* [19,49,52,58] (*n* = 4, 4%), *IL4* [49,50,51] (*n* = 3, 3%), *HRNR* [25,56,59] (*n* = 3, 3%) and *IL6* [50,51,53] (*n* = 3, 3%) loci, while evidence for possible association for relevant genes were examined by a total of two studies [19,42,44,50,51,53,54,60,61,62,63,64,65,66] (Table 1). Similarly, Asian studies primarily investigated SNPs mapped in the *FLG* [67,68,69,70,71,72,73,74,75,76,77,78,79,80,81,82,83,84] (*n* = 18, 18%), *IL4* [85,86,87,88,89,90,91] (*n* = 7, 7%) and *SPINK5* [67,92,93,94] (*n* = 4, 4%) genes, with the rest of the explored loci assessed by two or three studies [67,85,87,88,90,95,96,97,98,99,100,101,102,103,104,105,106,107,108,109,110,111,112] (Table 2).

### 3.2. Candidate Gene Approaches in Cases of European Ancestry

#### 3.2.1. FLG Gene

**Figure 2:**
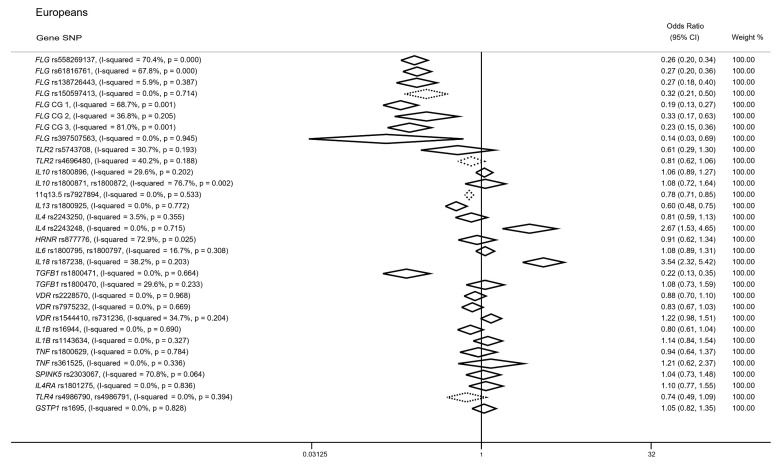
Association between single-nucleotide polymorphisms and atopic dermatitis susceptibility in patients of European descent. Dotted diamonds represent statistically significant small-study effects.

We confirmed all significant associations between the *FLG* loss-of-function (LOF) variants and increased AD risk from a total of 27 studies, incorporating 17,759 participants. Specifically, both *FLG* rs558269137 (OR (95% CI): 0.26 (0.20–0.34); I^2^ = 70.4%) and *FLG* rs61816761 (OR (95% CI): 0.27 (0.20–0.36); I^2^ = 67.8%) common alleles were associated with reduced AD risk from a total of 25 studies showing, however, a high heterogeneity (Figure 2; Supplementary Table S1). In addition, the rare alleles of the *FLG* rs138726443 (12 studies; *n* = 11,024), *FLG* rs150597413 (8 studies; *n* = 8356) and *FLG* rs397507563 (3 studies, *n* = 2780) SNPs yielded significant associations with higher risk of developing AD (OR (95% CI): 0.27 (0.18–0.40); I^2^ = 5.9%; OR (95% CI): 0.32 (0.21–0.50); I^2^ = 0.0%; OR (95% CI): 0.14 (0.03–0.69); I^2^ = 0.0%) (Figure 2; Supplementary Table S1).

The predominant role of the *FLG* locus in the disease onset, as well as the similar, *trans*-acting biological mechanism of the above variants [34], prompted us to investigate their effect under the spectrum of combined genotypes. Three combined genotypes (CGs) were identified from the included studies (CG1: rs558269137 and rs61816761; CG2: rs558269137, rs61816761 and rs138726443; CG3: rs558269137, rs61816761, rs138726443 and rs150597413); CG1 incorporated 5263 participants from 9 studies, CG2 included 2264 AD cases from 3 studies and CG3 was assessed in 5391 eczema patients from 4 studies. As expected, all CGs yielded significant associations (OR (95% CI): 0.19 (0.13–0.27); I^2^ = 68.7%; OR (95% CI): 0.33 (0.17–0.63); I^2^ = 36.8%; OR (95% CI): 0.23 (0.15–0.36); I^2^ = 81.0%) highlighting thus the contribution of the *FLG* locus in the disease predisposition, nevertheless reporting a high heterogeneity metric (Figure 2; Supplementary Table S1).

#### 3.2.2. TLR2 Gene

Our REM did not reveal a significant association between the *TLR2* rs5743708 polymorphism and disease onset from a total of 1257 participants (OR (95% CI): 0.61 (0.29–1.30); I^2^ = 30.7%). Similarly, the synthesis of evidence from three studies evaluating the *TLR2* rs4696480 SNP in 741 participants failed to establish a significant association with the risk of developing AD (OR (95% CI): 0.81 (0.62–1.06); I^2^ = 40.2%) (Figure 2; Supplementary Table S1).

#### 3.2.3. IL10 Gene

Comparably to the *TLR2* locus, our meta-analysis did not detect significant associations between the *IL10* rs1800896 variant and AD predisposition (OR (95% CI): 1.06 (0.89–1.27); I^2^ = 29.6%), using data from seven studies with 1703 participants (Figure 1). Notably, the *IL10* rs1800871 and *IL10* rs1800872 SNP were found in high LD SNPs (r^2^ > 0.9) and were consequently assessed as a single genotype; we did not observe significant associations between these variants and disease risk from a total of three studies (*n* = 1332; OR (95% CI): 1.08 (0.72–1.64); I^2^ = 76.7%) (Figure 2; Supplementary Table S1).

#### 3.2.4. 11. q13.5 Locus

The rs7927894 variant mapped to the 11q13.5 locus demonstrated a significant association with AD risk, based on five studies with 5506 participants (OR (95% CI): 0.78 (0.71–0.85); I^2^ = 0.0%) (Figure 2; Supplementary Table S1).

#### 3.2.5. IL13 Gene

Our systematic search identified four studies examining the *IL13* rs1800925 SNP (*n* = 931); synthesis of the available data depicted the association of the common rs1800925 SNP with reduced AD risk (OR (95% CI): 0.60 (0.48–0.75); I^2^ = 0.0%) (Figure 2; Supplementary Table S1).

#### 3.2.6. SNPs in IL4, IL18 and TGFB1 Genes

The common allele of *IL4* rs2243248 was significantly associated with a higher risk of developing AD, including data from two studies with a total of 503 participants (OR (95% CI): 2.67 (1.53–4.65); I^2^ = 0.0%). Contrastingly, the *IL4* rs2243250, assessed in three studies (*n* = 639) did not yield a significant outcome (OR (95% CI): 0.81 (0.59–1.13); I^2^ = 3.5%). Two studies further assessed SNPs mapped to the *IL18* (rs187238; *n* = 388) and *TGFB1* (rs1800471, rs1800470; *n* = 461) genes, providing significant associations with the exception of the *TGFB1* rs1800470 variant (Figure 2; Supplementary Table S1).

#### 3.2.7. SNPs in IL6, HRNR, VDR, IL1B, TNF, SPINK5, IL4RA, TLR4 and GSTP1 Genes

Two variants (*IL6* rs1800795 and rs1800797) were found in high LD and were therefore considered together during data synthesis; the meta-analysis from three studies (*n* = 1432) assessing these variants did not demonstrate significant association between the common allele and AD risk (OR (95% CI): 1.08 (0.89–1.31); I^2^ = 16.7%). Similarly, the *HRNR* rs877776 common allele was not associated with disease onset from a total of three studies incorporating 2158 participants (OR (95%CI): 0.91 (0.62–1.34); I^2^ = 72.9%). Additional investigation of putative risk loci through meta-analysis in two studies, including *VDR* rs2228570, rs7975232, *VDR* rs1544410 and rs731236 (r^2^ > 0.9), *IL1B* rs16944, *IL1B* rs1143634, *TNF* rs1800629, *TNF* rs361525, *SPINK5* rs2303067, *IL4RA* rs1801275, *TLR4* rs4986790 and rs4986791 (r^2^ > 0.9) and *GSTP1* rs1695, gave null results (Figure 2; Supplementary Table S1).

### 3.3. Candidate Gene Approaches in Cases of Asian Ancestry

#### 3.3.1. FLG Gene

**Figure 3:**
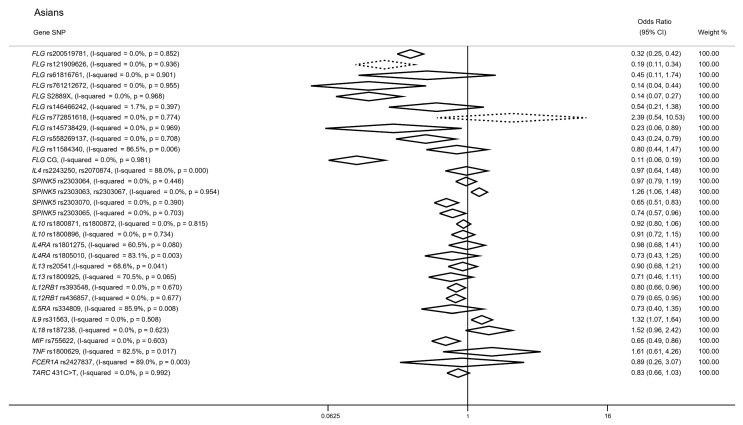
Association between single-nucleotide polymorphisms and atopic dermatitis susceptibility in patients of Asian descent. Dotted diamonds represent statistically significant small-study effects.

Similarly to the studies assessing population of European descent, we validated significant associations between *FLG* LOF variants and AD risk in participants of Asian ancestry. In particular, the *FLG* rs200519781 frameshift variant was associated with increased AD risk from a total of 14 studies (*n* = 7704; OR (95% CI): 0.32; (0.25–0.42); I^2^ = 0.0%). As far as *FLG* rs121909626 (9 studies; *n* = 4054) and *FLG* rs761212672 (6 studies; *n* = 2092) SNPs are concerned, both yielded significant associations with higher eczema risk (OR (95% CI): 0.19 (0.11–0.34); I^2^ = 0.0%; OR (95% CI): 0.14 (0.04–0.44); I^2^ = 0.0%). Our REM highlighted two additional *FLG* variants (rs145738429, 5 studies, *n* = 1857; S2889X, 6 studies, *n* = 1736) associated with increased AD risk (OR (95% CI): 0.23 (0.06–0.89); I^2^ = 0.0%; OR (95% CI): 0.14 (0.07–0.27); I^2^ = 0.0%). Notably, the *FLG* rs558269137 variant was further associated in participants of Asian ancestry from a total of two studies including 1382 cases (OR (95% CI): 0.43 (0.24–0.79); I^2^ = 0.0%) (Figure 3; Supplementary Table S2).

Despite the abundance of statistically significant associations between *FLG* variants and increased AD risk in both Europeans and Asians, several variants, including *FLG* rs61816761 (7 studies; *n* = 3085), *FLG* rs146466242 (5 studies; *n* = 1930), *FLG* rs772851618 (5 studies; *n* = 1857) and *FLG* rs11584340 (2 studies; *n* = 855), failed to provide significant signals (OR (95% CI): 0.45 (0.11–1.74); I^2^ = 0.0%; OR (95% CI): 0.54 (0.21–1.38); I^2^ = 1.7%; OR (95% CI): 2.39 (0.54–10.53); I^2^ = 0.0%; OR (95% CI): 0.80 (0.44–1.47); I^2^ = 86.5%) (Figure 3; Supplementary Table S2).

Regarding Asians, we identified one CG from the included studies (CG: rs200519781, rs121909626, rs761212672, rs145738429, S2889X, rs61816761, rs146466242 and rs772851618); CG incorporated 877 participants from three studies and yielded a significant association (OR (95% CI): 0.11 (0.06–0.19); I^2^ = 0.0%) (Figure 3; Supplementary Table S2).

#### 3.3.2. IL4 Gene

Two variants mapped to the *IL4* gene (rs2243250, rs2070874) were found in high LD (r^2^ > 0.9) and were thus examined together from a total of seven studies (*n* = 2454). Our REM did not demonstrate a significant association (OR (95% CI): 0.97 (0.64–1.48); I^2^ = 88.0%) (Figure 3; Supplementary Table S2).

#### 3.3.3. SPINK5 Gene

Comparably to *IL4* variants, two *SPINK5* variants (rs2303063, rs2303067) were found in high LD (r^2^ > 0.9) and were thus examined together from a total of three studies (*n* = 1280). However, we found that the above locus was significantly associated with AD risk (OR (95% CI): 1.26 (1.06–1.48); I^2^ = 0.0%). Relevant, independent variants mapped to the SPINK5 gene yielded additional significant signals, with the examples of *SPINK5* rs2303070 (3 studies, *n* = 741; OR (95% CI): 0.65 (0.51–0.83); I^2^ = 0.0%) and *SPINK5* rs2303065 (two studies, *n* = 546; OR (95% CI: 0.74 (0.57–0.96); I^2^ = 0.0%). Nevertheless, data synthesis from four studies (*n* = 930) regarding the *SPINK5* rs2303064 SNP showed no evidence for an association with AD predisposition in the Asian population (OR (95% CI): 0.97 (0.79–1.19); I^2^ = 0.0%) (Figure 3; Supplementary Table S2).

#### 3.3.4. SNPs in IL10, IL4RA and IL13 Genes

Three studies assessed the association of *IL10* rs1800896, *IL10* rs1800871 rs1800872 (r^2^ > 0.9), *IL4RA* rs1801275, *IL4RA* rs1805010 and *IL13* rs20541, while two studies assessed the *IL13* rs1800925 in AD Asians. Our REM gave null results in all above cases (Figure 3; Supplementary Table S2).

#### 3.3.5. SNPs in IL12RB1, IL9 and MIF Genes

Considering the *IL12RB1* rs393548 and rs436857 variants, two studies assessed their association with disease onset (*n* = 1339, *n* = 1326, respectively). Our meta-analysis yielded statistically significant associations in both cases (OR (95% CI): 0.80 (0.66–0.96); I^2^ = 0.0%; OR (95% CI): 0.79 (0.65–0.95); I^2^ = 0.0%) (Figure 3; Supplementary Table S2). In addition, synthesis of the available data derived from two studies regarding the *IL9* rs31563 (*n* = 1391) and *MIF* rs755622 (*n* = 649) SNPs provided significant associations with AD risk (OR (95% CI): 1.32 (1.07–1.64); I^2^ = 0.0%; OR (95% CI): 0.65 (0.49–0.86); I^2^ = 0.0%) (Figure 3; Supplementary Table S2).

#### 3.3.6. SNPs in IL5RA, IL18, TNF, FCER1A and TARC Genes

Lastly, two studies examined the association between variants mapped to the *IL5RA* (rs334809), *TNF* (rs1800629), *FCER1A* (rs2427837) and *TARC* (431C > T) genes, generating non-significant results (Figure 3; Supplementary Table S2). Remarkably, despite the significant association in cases of European descent, the *IL18* rs187238 was not associated with AD risk in the Asian population (OR (95% CI): 1.52 (0.96–2.42); I^2^ = 0.0%) (Figure 3; Supplementary Table S2).

Harbord’s modified test identified small-study effect biases in six cases. Specifically, we found a small-study effect bias in the REM of *FLG* rs150597413 (*p*-value = 0.071), *TLR2* rs4696480 (*p*-value = 0.006), 11q13.5 rs7927894 (*p*-value = 0.062) and *TLR4* rs4986790, rs4986791 (*p*-value = 0.019) in European cases (Figure 2), while significant biases were found in the *FLG* rs121909626 (*p*-value = 0.049) and *FLG* rs772851618 (*p*-value = 0.021) in Asians (Figure 3).

## 4. Discussion

Here, we performed a systematic review and meta-analysis of all available evidence considering the genetic predisposition to AD in participants of both European and Asian ancestry. Despite the expanding list of risk loci identified through genome-wide scans in AD, our REM shed light upon new risk loci mapped to the *IL18* and *TGFB1* genes in Europeans (Figure 2), while the contribution of *IL12RB1* and *MIF* loci in AD predisposition in Asians was additionally characterized through our analysis, revealing results not previously reported by genome-wide scans (Figure 3). We thus provide novel insights into the genetic architecture of the disease, highlighting perturbed biological pathways that could be further implicated in the development of new therapeutic mechanisms.

Several of our findings have been previously discovered by GWASs in both participants of European and Asian ancestries. In particular, the present meta-analysis confirms the pivotal role of the *FLG* locus in disease susceptibility, as LOF mutations disrupt the production of the filaggrin protein in the stratum corneum. The impaired barrier function facilitates a loss of water and pH increase, as well as the entry of irritants and allergens, contributing to the onset and exacerbation of allergic reactions [113]. The consistency of these findings across European and Asian populations highlights the universal importance of *FLG* in maintaining skin homeostasis and its relevance in AD [114]. The presence of significant heterogeneity concerning *FLG* rs558269137 and rs61816761 in European population was also observed in a prior meta-analysis of *FLG* polymorphisms in AD [115], which could be attributed to several demographic factors and environmental exposures that mediate the increased atopic eczema risk [113]. We further validate the 11q13.5 rs7927894 SNP as a risk locus, a variant localized within a non-coding genomic region. Notably, the above variant has been additionally implicated in Crohn’s disease and deregulated epithelial function, thus potentially contributing to the pathogenic mechanisms underlying AD [55]. The cytokine cluster located at 5q31, harboring both *IL4* and *IL13*, represents a prominent risk locus implicated in the pathogenesis of the disease and has been extensively linked with AD through several GWAS-derived variants. Here, we identified the *IL13* rs1800925 SNP, a regulatory variant that enhances the activity of the *IL13* promoter in human Th2 lymphocytes, thereby augmenting the risk of allergic disorders [10]. Remarkably, this particular variant further exhibits a significant association with cutaneous T-cell lymphoma [116], a malignancy that has been observed to manifest in some cases of AD patients following the administration of dupilumab [117], an anti-IL4RA biological drug that inhibits both *IL4* and *IL13* signaling [11]. Another significantly associated variant that belongs to the 5q31 locus is the *IL4* rs2243248 SNP, where our REM reported the protective effect of the minor rs2243248 G allele in AD risk, similar to asthma [9]. Relevant, previously identified risk variants refer to the *SPINK5* nonsynonymous variants that disrupt the function of serine protease inhibitors in the integrity of the epidermal barrier, as well as the *IL9* rs31563 variant that exhibits a regulatory role in *IL9* expression, a cytokine known to contribute to B cell isotype switching from IgM to IgE synthesis [102].

Despite the prominence of GWAS outcomes as the gold standard in genetic association studies, there are still certain risk loci that have not been yet identified. We further report four novel loci, two belonging to Europeans (Figure 2) and two Asian-specific (Figure 3). In particular, we detected a significant association between the *IL18* rs187238 polymorphism and AD risk in European cases (Figure 2); this variant, residing within the *IL18* promoter region, supports the modulation of the binding affinity of transcription factors and thus participates in the dysregulated Th2 response [118]. Our second novel addition in Europeans is the *TGFB1* rs1800471 missense variant (Figure 2) that significantly alters the inhibitory action of TGFB1 protein molecule during allergic reactions [119].

In addition, the promoter *IL12RB1* rs393548 and rs436857 variants were significantly associated with AD risk in Asians (Figure 3); the *IL12RB1* receptor subunit is involved in the modulation of the *IL12*-dependent inhibition of IgE synthesis and Th2 cell function. Consequently, a potential decrease of the *IL12RB1* expression perturbs the above inhibitory activity [101], thus leading to the dysregulation of downstream immune responses and potentially contributing to allergic reactions. Finally, our findings indicate a significant association between the *MIF* rs755622 C allele and increased AD susceptibility (Figure 3); the rare C allele exhibits enhanced promoter activity relative to the common G allele, leading to differential *MIF* expression levels [103]. Studies conducted on mouse models of AD have demonstrated that *MIF* holds a significant role in the development of AD-related immune dysregulation through the induction of a type 2 immune response, as well as through fostering the recruitment of eosinophils in the cutaneous inflammation [120].

However, our study displays some constraints. Despite the assessment of small-study effect bias through the Harbord’s modified test and the identification of six significant cases, the majority of studies included in our synthesis incorporated a relatively small sample size and are thus susceptible to biases [121]. In addition, discrepancies between the available data derived from the included studies, with the examples of disease activity, age of onset and relevant clinical information, restricted further stratification and subgroup examination in our analysis. The predominant role of the exposome in the AD predisposition [113] and the inclusion of such environmental risk factors in multivariate analyses is of paramount importance to gain a holistic understanding of AD etiopathology and thus unveil novel therapeutic targets.

In conclusion, we conducted a systematic review and meta-analysis of all available data regarding the genetic predisposition to AD in participants of both European and Asian ancestry. We report four novel risk loci that have not been previously shown by GWAS, partially contributing to the elucidation of the genetic architecture of the disease. By exploring data from both ethnic groups, our study endeavors to provide a broader understanding and enhance the applicability of our findings across diverse populations. High-throughput investigation of putative risk loci in AD [122,123] could further unveil their functional role in the disease predisposition. In addition, the incorporation of clinical information for each included participant, as well as the vast number of environmental exposures associated with increased AD risk, shall facilitate the interpretation of disease-associated interactions and form the framework for precision medicine.

## Figures and Tables

**Table 1:** Studies of European descent identified through our systematic search.

Study, Year	Rs ID	Sample Size
Vardar Acar N et al., 2020 [14]	rs558269137, rs61816761, rs138726443, rs150597413	189
Jurakic Toncic R et al., 2020 [15]	rs558269137, rs61816761, rs138726443	150
González-Tarancón R et al., 2020 [16]	rs558269137, rs61816761, rs138726443, CG2	214
Gimalova GF et al., 2016 [17]	rs558269137, rs61816761	564
Woźniak M et al., 2016 [18]	rs558269137, rs61816761, CG1	121
Trzeciak M et al., 2016 [19]	rs558269137, rs61816761, rs1800925, rs187238	275
Trzeciak M et al., 2016 [20]	rs558269137	256
Komova EG et al., 2014 [21]	rs558269137, rs61816761, rs138726443, rs150597413	230
Ballardini N et al., 2013 [22]	rs558269137, rs61816761, rs138726443, CG2	1854
Ercan H et al., 2013 [23]	rs61816761	99
Mlitz V et al., 2012 [24]	rs558269137, rs61816761, rs138726443, CG2	196
O’Regan GM et al., 2010 [25]	rs558269137, rs61816761, rs138726443, rs150597413, CG3, rs7927894, rs877776	1511
Greisenegger E et al., 2010 [26]	rs558269137, rs61816761, rs138726443, rs150597413, CG3	864
Gao PS et al., 2009 [27]	rs558269137, rs61816761, CG1	435
Brown SJ et al., 2008 [28]	rs558269137, rs61816761, rs138726443, rs150597413, rs397507563	811
Brown SJ et al., 2008 [29]	rs558269137, rs61816761, rs138726443, rs150597413, rs397507563	1221
Giardina E et al., 2008 [30]	rs558269137, rs61816761	388
Weidinger S et al., 2008 [31]	rs558269137, rs61816761, rs138726443, rs150597413, CG3	3099
Rogers AJ et al., 2007 [32]	rs558269137, rs61816761, CG1	646
Lerbaek A et al., 2007 [33]	rs558269137, rs61816761, CG1	215
Sandilands A et al., 2007 [34]	rs558269137, rs61816761, rs138726443, rs150597413, rs397507563	924
Weidinger S et al., 2007 [35]	rs558269137, rs61816761, CG1	526
Marenholz I et al., 2006 [36]	rs558269137, rs61816761, CG1	507
Stemmler S et al., 2007 [37]	rs558269137, rs61816761, CG1	1078
Barker JN et al., 2007 [38]	rs558269137, rs61816761, CG1	1626
Palmer CN et al., 2006 [39]	rs558269137, rs61816761, CG1	241
Dêbiñska A et al., 2020 [40]	CG3, rs7927894	188
Can C et al., 2017 [41]	rs5743708, rs4696480	139
Salpietro C et al., 2011 [42]	rs5743708, rs4696480, rs4986790, rs4986791	337
Galli E et al., 2010 [43]	rs5743708	249
Oh DY et al., 2009 [44]	rs5743708, rs4696480, rs4986790, rs4986791	265
Niebuhr M et al., 2008 [45]	rs5743708	19
Levchenko L Yu et al., 2013 [46]	rs5743708	131
Ahmad-Nejad et al., 2003 [47]	rs5743708	117
Esposito S et al., 2015 [48]	rs1800896, rs1800872	223
Lesiak A et al., 2014 [49]	rs1800896, rs1800925, rs2243250	136
Kayserova J et al., 2012 [50]	rs1800896, rs1800871, rs1800872, rs2243250, rs2243248, rs1800795, rs1800797, rs1801275	197
Stavric K et al., 2012 [51]	rs1800896, rs1800871, rs1800872, rs2243250, rs2243248, rs1800471, rs1800470, rs1800795, rs1800797, rs16944, rs1143634, rs1800629, rs361525, rs1801275	367
Lesiak A et al., 2011 [52]	rs1800896, rs1800925	367
Reich K et al., 2003 [53]	rs1800896, rs1800795, rs16944, rs1143634, rs1800629, rs361525	308
Arkwright PD et al., 2001 [54]	rs1800896, rs1800471, rs1800470	118
Ponińska JK et al., 2017 [55]	rs7927894	810
Greisenegger EK et al., 2013 [56]	rs7927894, rs877776	518
Marenholz I et al., 2011 [57]	rs7927894	2485
Hummelshoj T et al., 2003 [58]	rs1800925	159
Dêbiñska A et al., 2022 [59]	rs877776	188
Trzeciak M et al., 2010 [60]	rs187238	113
Kılıç S et al., 2016 [61]	rs2228570, rs7975232, rs1544410, rs731236	138
Heine G et al., 2013 [62]	rs2228570, rs7975232, rs1544410, rs731236	530
Dežman K et al., 2017 [63]	rs2303067	405
Fölster-Holst R et al., 2005 [64]	rs2303067	569
Vavilin VA et al., 2003 [65]	rs1695	325
Safronova OG et al., 2003 [66]	rs1695	274

Abbreviations: CG, Combined Genotype.

**Table 2:** Studies of Asian descent identified through our systematic search.

Study, Year	Rs ID	Sample Size
Kim BJ et al., 2019 [67]	rs200519781, rs146466242, rs2303064, rs2303070, rs2303065, rs393548, rs436857, rs31563, rs334809	325
Sasaki T et al., 2014 [68]	rs200519781, rs121909626, rs761212672, rs145738429, rs61816761, rs146466242, rs772851618	721
Meng L et al., 2014 [69]	rs200519781	1988
Lee DE et al., 2013 [70]	rs200519781	175
Chen H et al., 2011 [71]	rs200519781, rs61816761	865
Zhang H et al., 2011 [72]	rs200519781	353
Osawa R et al., 2010 [73]	rs200519781, rs121909626, rs761212672, rs145738429, S2889X, rs61816761, rs146466242, rs772851618, CG	306
Nomura Y et al., 2010 [74]	rs200519781, rs121909626, rs761212672, rs145738429, S2889X, rs61816761, rs146466242, rs772851618, CG	307
Ma L et al., 2010 [75]	rs200519781	329
Nemoto-Hasebe I et al., 2009 [76]	rs200519781, rs121909626, rs761212672, rs145738429, S2889X, rs61816761, rs146466242, rs772851618, CG	271
Nomura T et al., 2009 [77]	rs200519781, rs121909626, rs761212672, rs145738429, S2889X, rs61816761, rs772851618	252
Nomura T et al., 2008 [78]	rs200519781, rs121909626, rs761212672, S2889X	235
Enomoto H et al., 2008 [79]	rs200519781, rs121909626	1299
Nomura T et al., 2007 [80]	rs200519781, rs121909626	299
Ching GK et al., 2009 [81]	rs121909626, S2889X, rs558269137, rs61816761	365
Zhong WL et al., 2016 [82]	rs558269137	1017
Kim SY et al., 2013 [83]	rs11584340	527
Wang IJ et al., 2011 [84]	rs11584340	328
Hua L et al., 2021 [85]	rs2243250, rs2070874, rs1801275, rs1805010, rs20541	597
Shang H et al., 2016 [86]	rs2243250, rs2070874	182
Hussein YM et al., 2016 [87]	rs2243250, rs2070874, rs1805010	100
Gharagozlou M et al., 2015 [88]	rs2243250, rs2070874, rs1801275	228
Hussein YM et al., 2014 [89]	rs2243250, rs2070874	206
Tanaka K et al., 2001 [90]	rs2243250, rs2070874, rs1805010	424
Kawashima T et al., 1998 [91]	rs2243250, rs2070874	425
Morizane S et al., 2018 [92]	rs2303064, rs2303063, rs2303067, rs2303070	107
Zhao LP et al., 2012 [93]	rs2303064, rs2303063, rs2303067, rs2303070	341
Kato A et al., 2003 [94]	rs2303064, rs2303063, rs2303067, rs2303065	234
Bin Huraib G et al., 2018 [95]	rs1800871, rs1800872, rs1800896, rs1800629	315
Behniafard N et al., 2018 [96]	rs1800871, rs1800872, rs1800896	229
Sohn MH et al., 2007 [97]	rs1800871, rs1800872, rs1800896	416
Miyake Y et al., 2013 [98]	rs1801275	823
Miyake Y et al., 2011 [99]	rs20541, rs1800925	1270
Miyake Y et al., 2011 [100]	rs20541, rs1800925	533
Takahashi N et al., 2005 [101]	rs393548, rs436857	1040
Namkung JH et al., 2011 [102]	rs31563	1090
Kim JS et al., 2016 [103]	rs755622	258
Ma L et al., 2013 [104]	rs755622	391
Miyake Y et al., 2015 [105]	rs334809	1318
Kato T et al., 2009 [106]	rs187238	264
Osawa K et al., 2007 [107]	rs187238	121
Behniafard N et al., 2012 [108]	rs1800629	226
Zhou J et al., 2012 [109]	rs2427837	380
Park KY et al., 2011 [110]	rs2427837	231
Tsunemi Y et al., 2004 [111]	431C > T	351
Sekiya T et al., 2003 [112]	431C > T	306

Abbreviations: CG, Combined Genotype.

## Data Availability

All data are available from the corresponding author upon reasonable request.

## Supplementary Materials

The following supporting information can be downloaded at: https://www.mdpi.com/article/10.3390/genes14071456/s1, Table S1. Association between single nucleotide polymorphisms and atopic dermatitis susceptibility in patients of European descent. Table S2. Association between single nucleotide polymorphisms and atopic dermatitis susceptibility in patients of Asian descent.
